# Enhanced Endoscope is Associated With Success Rates in B2‐ Endoscopic Ultrasound‐Guided Hepaticogastrostomy

**DOI:** 10.1002/deo2.70279

**Published:** 2026-01-28

**Authors:** Yoshitaro yamamoto, Kazuo Hara, Nozomi Okuno, Shin Haba, Takamichi Kuwahara, Hiroki Koda, Minako Urata, Takashi Kondo, Keigo Oshiro, Tomoki Ogata, Ren Kuwabara

**Affiliations:** ^1^ Department of Gastroenterology Aichi Cancer Center Hospital Nagoya Japan

**Keywords:** endoscopic ultrasound‐guided biliary drainage, endoscopic ultrasound‐guided hepaticogastrostomy, forward‐viewing (FV), interventional EUS, oblique‐viewing (OV)

## Abstract

**Objective:**

Endoscopic ultrasound (EUS)‐guided hepaticogastrostomy (HGS) in intrahepatic bile duct segment 3 (B3) is widely used for biliary drainage. Post‐puncture procedures are easy to perform in intrahepatic bile duct segment 2 (B2), but using a conventional oblique‐viewing (OV) scope (GF‐UCT260) may result in transesophageal puncture. In this study, we compared the safety and efficacy of B2 puncture using a conventional OV scope, a novel OV scope (EG‐740UT), and a forward‐viewing (FV) scope (TGF‐UC260J).

**Methods:**

This single‐center retrospective study investigated 319 consecutive patients in whom B2‐EUS‐HGS was attempted using an OV or FV between January 2017 and March 2024 at Aichi Cancer Center.

**Results:**

In B2‐EUS‐HGS, the use of enhanced endoscopes (TGF‐UC260J and EG‐740UT) resulted in a relatively high technical success rate of 93.6% (205/219) and an overall clinical success rate of 97.1% (199/205). The enhanced endoscope group demonstrated a significantly higher technical success rate (*p* < 0.001) compared to the conventional endoscope group. No significant differences were observed between the two groups in terms of overall clinical success rate (*p* = 0.128) and early adverse event rate (*p* = 0.461).

**Conclusions:**

B2‐EUS‐HGS using either an FV or novel OV scope showed comparable safety with a high technical and overall clinical success rate. The use of an FV or novel OV scope seems to be a suitable strategy for performing B2‐EUS‐HGS.

**Clinical Trial Registration:**

Study/trial registration and registration number were not applicable (N/A), as this study was a retrospective analysis using anonymized data.

## 1 Introduction

Biliary drainage (BD) using endoscopic ultrasound (EUS) has gained widespread recognition as a preferred method for treating biliary obstruction, particularly in cases where endoscopic retrograde cholangiopancreatography (ERCP) is not a viable option [1, 2, 3].

Among the various techniques, EUS‐guided hepaticogastrostomy (HGS) has recently been used as a primary drainage method [4]. Few devices dedicated to EUS‐HGS have been reported, but serious adverse events (AEs) sometimes occur. The incidence of AEs appears to be influenced by the proficiency and experience level of the performing endoscopist [5, 6]. Previous reports recommend that EUS‐guided BD should be performed by an experienced endoscopist who has acquired substantial expertise in various advanced endoscopic procedures [7, 8, 9, 10]. Specifically, the endoscopist should have extensive hands‐on experience in ERCP, EUS for diagnostic screening, and EUS‐guided fine‐needle aspiration (FNA) or related interventions targeting the biliopancreatic region. This expertise is essential to ensure technical success, minimize complications, and optimize patient outcomes. Due to the risk of AEs [11, 12] with intrahepatic bile duct segment 2 (B2) puncture, intrahepatic bile duct segment 3 (B3) puncture is generally preferred [13]. However, we have previously reported that compared to B3, B2 puncture is technically easier for performing guidewire insertion, dilation, and forward stenting, making B2‐EUS‐HGS safer and more feasible [14]. At our institution, B2 puncture is the first‐choice strategy for EUS‐HGS. We have reported the use of a novel oblique‐viewing (OV) scope (EG‐740UT; Fujifilm, Tokyo, Japan) or a forward‐viewing (FV) scope (TGF‐UC260J; Olympus, Tokyo, Japan) to more easily perform B2 puncture, making B2‐EUS‐HGS safer and more effective [15, 16]. However, no reports have compared the conventional OV scope (GF‐UCT260; Olympus, Tokyo, Japan), novel OV scope, and FV scope for B2‐EUS‐HGS. This study was conducted as a retrospective analysis to assess the safety and effectiveness of B2‐EUS‐HGS. The evaluation specifically focused on procedures performed using three different EUS scopes, aiming to determine their clinical outcomes, procedural success rates, and potential complications.

## 2 Methods

### 2.1 Patients

**FIGURE 1:**
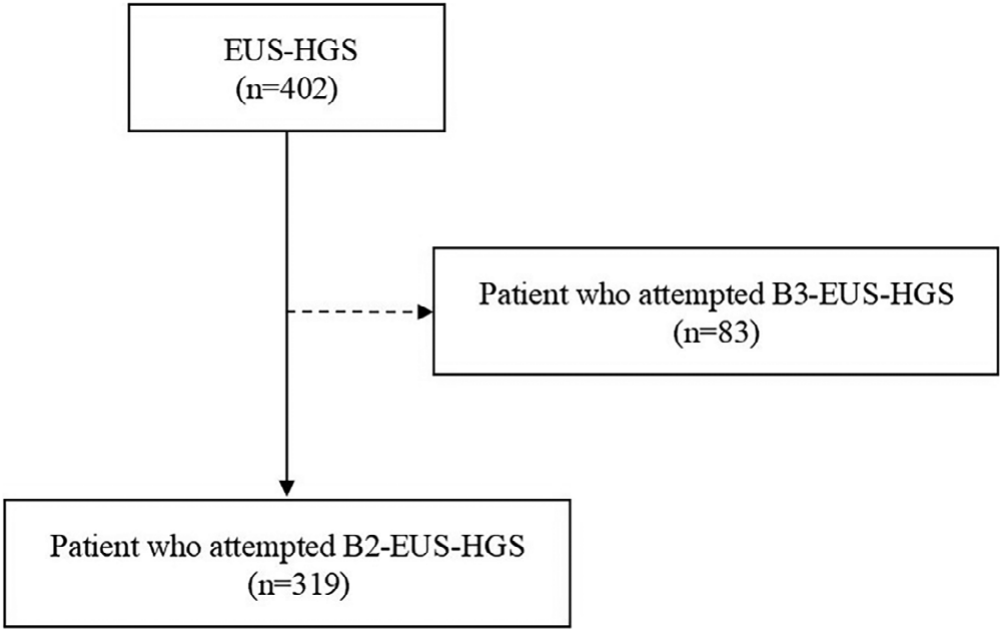
Flow in this study. B2, bile duct segment 2; B3, bile duct segment 3; EUS‐HGS, endoscopic ultrasound‐guided hepaticogastrostomy.

Between January 2017 and March 2024, a total of 402 consecutive patients underwent EUS‐HGS at Aichi Cancer Center. Among them, 319 patients specifically underwent B2‐EUS‐HGS utilizing one of three different EUS scopes: the conventional OV scope (GF‐UCT260), the novel OV scope (EG‐740UT), or either the OV scope or FV scope (TGF‐UC260J). These 319 patients were prospectively enrolled in the study, and their clinical data, including procedural details, success rates, and potential complications, were retrospectively collected and analyzed (Figure 1).

### 2.2 Conventional OV Scope and Novel OV Scope and FV Scope

At our institution, the GF‐UCT260 was used for B2‐EUS‐HGS starting in January 2017. After 100 B2‐EUS‐HGS procedures had been performed using the GF‐UCT260, the TGF‐UC260J was used in 106 B2‐EUS‐HGS procedures starting in January 2020. Subsequently, between September 2021 and March 2024, B2‐EUS‐HGS was performed in 113 patients using the EG‐740UT.

**FIGURE 2:**
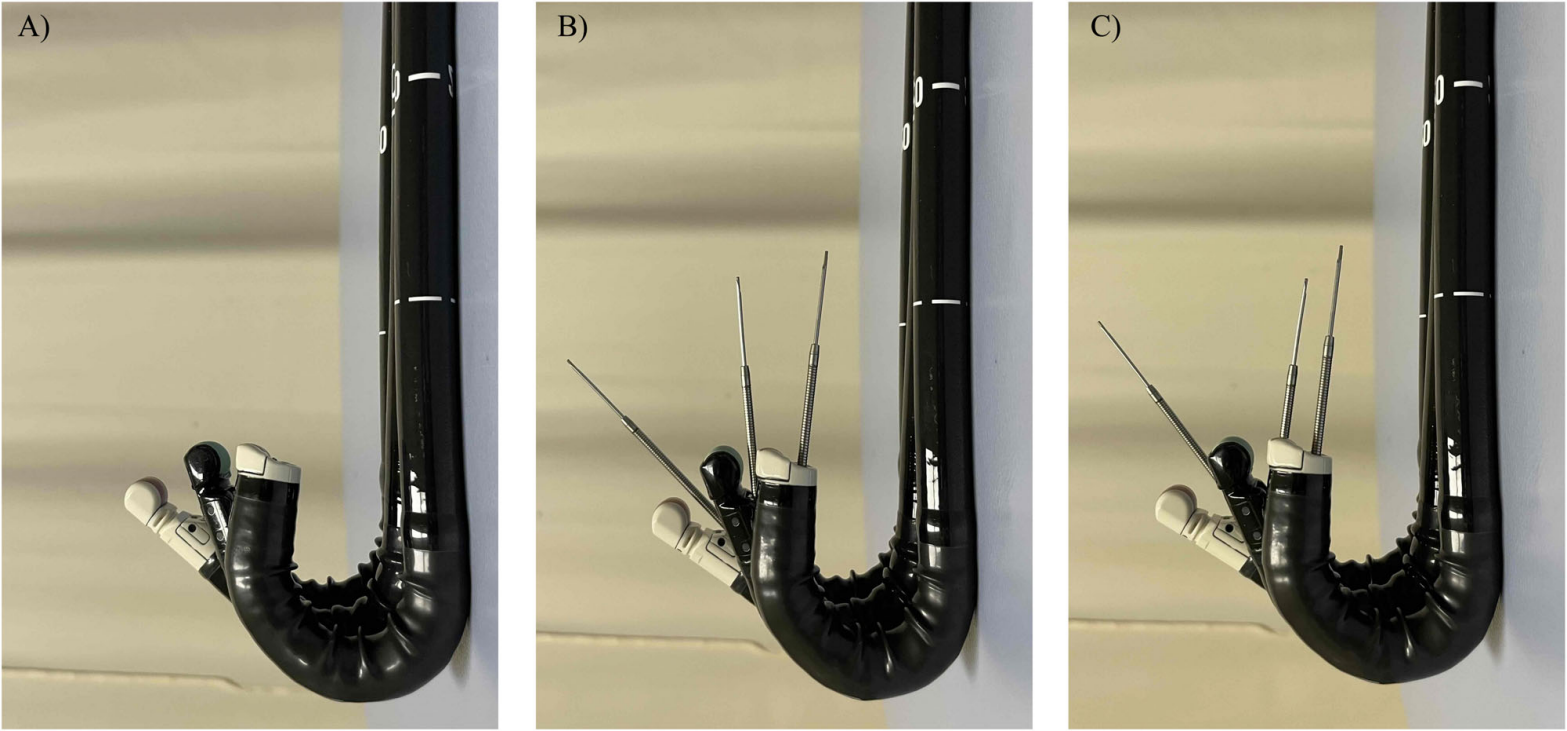
Comparison of the TGF‐UC260J, EG‐740UT, and GF‐UCT260 scopes. (A) Full‐up angulation of the TGF‐UC260J (front), EG‐740UT (middle), and GF‐UCT260 (back). (B) Full‐up angulation of the TGF‐UC260J with a 19‐G fine needle aspiration (FNA) needle, and full‐up angulation without use of the EG‐740UT and GF‐UCT260 elevators with a 19‐G FNA needle. (C) Full‐up angulation of the TGF‐UC260J with a 19‐G FNA needle, and full‐up angulation and maximum elevation of the EG‐740UT and GF‐UCT260 elevators with a 19‐G FNA needle.

Figure 2 presents a detailed comparison of three different EUS scopes—TGF‐UC260J, EG‐740UT, and GF‐UCT260—alongside a 19‐G FNA needle (EZ Shot 3Plus; Olympus, Tokyo, Japan). The respective up‐angulation ranges are 180° for the TGF‐UC260J, 150° for the EG‐740UT, and 130° for the GF‐UCT260 (Figure 2A). Among the three scopes, the TGF‐UC260J has the largest up‐angulation in its natural state with the FNA needle loaded (Figure 2B), but this scope does not have an elevator function and so is not significantly different from the EG‐740UT when the FNA needle is loaded, and the needle is maximally up (Figure 2C).

### 2.3 Technical Tips for B2‐EUS‐HGS

In our previous research, we reported that in patients with severe ascites, performing ascitic fluid drainage before EUS‐HGS can significantly reduce the risk of developing severe peritonitis. This preemptive intervention helps prevent complications associated with the leakage of bile or intestinal contents into the peritoneal cavity [17]. B2‐EUS‐HGS procedure, utilizing either an FV or OV scope, is carried out according to the following step‐by‐step protocol. At our institution, B2 puncture is the first choice. If the B2 puncture proves difficult, we then change to the B3 puncture. After the introduction of the enhanced endoscope, either the TGF‐UC260J or EG‐740UT was principally used. When B2 puncture was attempted but found to be difficult, the approach was changed to B3 puncture without switching the scope itself. The choice of needle for B2‐EUS‐HGS is determined based on the diameter of the targeted B2 bile duct. A 19‐G needle (EZ Shot3 Plus; Olympus, Tokyo, Japan) is used when the bile duct diameter is 2.5 mm or greater, whereas a 22‐G needle (Expect Slimline; Boston Scientific, Natick, MA, USA) is selected when the diameter is less than 2.5 mm. When using the TGF‐UC260J or GF‐UCT260, the scopes are connected to an ultrasound system (EU‐ME2; Olympus, Tokyo, Japan). When using the EG‐740UT, imaging is performed with a different ultrasound system (SU‐1; Fujifilm, Tokyo, Japan). Before puncturing the bile duct with a needle, a connector (RADIFOCUS Hemostasis Valve II; Terumo, Tokyo, Japan) is attached to the needle. This connector is prefilled with contrast medium to enhance fluoroscopic visibility. For the 19‐G needle, a 0.025‐inch guidewire (M‐Through; Asahi Intecc, Aichi, Japan) is preloaded. For the 22‐G needle, a rotational hemostatic valve (0.096; Abbott, Tokyo, Japan) is preloaded with a 0.018‐inch guidewire (Fielder18; Olympus, Tokyo, Japan) and filled with contrast medium before use. This assembly is then securely connected to the needle, ensuring precise access to the narrow bile duct while minimizing the risk of procedural complications.

### 2.4 Endpoint

The primary endpoint of this study was to evaluate the technical success rate of B2‐EUS‐HGS. The secondary endpoint was to assess the incidence of procedure‐related AEs, including complications such as stent migration, bile leakage, and infection.

### 2.5 Definitions and Statistical Analysis

Enhanced endoscopes (TGF‐UC260J and EG‐740UT) are defined as scopes that allow puncture at highly angulated orientations. Conventional endoscopes (GF‐UCT260) are defined as scopes used for puncture at standard angulation.

An endoscopist with experience of 20 or more EUS‐HGS procedures was defined as an expert, while those with fewer than 20 cases were defined as non‐experts [18].

Technical success has been defined as the successful placement of at least one stent or catheter in the intended location of the bile duct [19]. Clinical success has been defined as a ≥50% reduction or normalization of total bilirubin within 14 days of BD for cases with jaundice and a sufficient reduction (which is evaluated clinically on a case‐by‐case basis) or normalization of target liver enzymes within 14 days for cases receiving drainage for elevated levels of other liver enzymes due to biliary obstruction [20].

Procedure time was defined as the duration from the initial puncture of the bile duct to the completion of stent deployment during the EUS‐HGS procedure. BD was categorized into two main types: primary drainage, performed as the initial method of biliary decompression, and salvage drainage, carried out following the failure or inadequacy of prior drainage attempts.

Primary drainage was defined as the initial attempt at biliary decompression, representing the first‐line drainage procedure performed in the clinical course. In contrast, salvage drainage referred to the use of EUS‐HGS as a rescue technique, employed after prior BD methods—such as percutaneous transhepatic BD (PTBD), transpapillary drainage, or EUS‐guided choledochoduodenostomy (EUS‐CDS)—had been attempted but failed to achieve adequate BD.

The method of classifying the severity of ascites based on CT is important for assessing the spread of ascites and its effects on organs. The severity of ascites was categorized as mild, moderate, or severe. Mild ascites was defined as the presence of fluid limited to specific anatomical spaces, such as the pouch of Douglas (rectosigmoid fossa) or Morison's pouch (the space between the liver and right kidney), without evidence of generalized or extensive peritoneal fluid accumulation.

This indicates that the ascites is limited and not spreading. Moderate ascites was defined as ascites intermediate between mild and severe. This is the stage where the ascites is spreading but does not yet cover the entire abdomen. Severe ascites was defined as ascites covering the abdominal organs.

AEs associated with endoscopic procedures were assessed based on the criteria established by the American Society for Gastrointestinal Endoscopy guidelines, which provide standardized definitions and severity grading for procedure‐related complications [20]. Descriptive statistics were presented as medians with ranges or means with standard deviations for continuous variables, and as frequencies for categorical variables. Comparative analyses of quantitative variables were conducted using Fisher's exact test or the Chi‐square test, as appropriate. A *p*‐value of less than 0.05 was considered to indicate statistical significance. Continuous variables were expressed as mean values. All statistical analyses were performed using EZR version 1.68 (Saitama Medical Center, Jichi Medical University, Saitama, Japan).

## 3 Results

Table 1 provides a summary of the patient demographics and clinical characteristics. A total of 319 patients were analyzed and categorized into two groups based on the type of scope used (conventional endoscope vs. enhanced endoscope). No duplicate patients were included in the 319 cases. The following variables were compared between the two groups: age, sex, primary disease, indication for EUS‐HGS, presence of ascites, and whether ascites drainage was performed before EUS‐HGS. A significant difference was observed in the presence of ascites (*p* = 0.017), whereas no significant differences were found in the other variables.

**TABLE 1:** Patient's characteristics.

Characteristics	All (*n* = 319)	Conventional endoscope (*n* = 100)	Enhanced endoscope (*n* = 219)	*p*‐Value
Median age (y, range)	68 (24–91)	67 (40–87)	69 (24–91)	0.807
Sex (male/female)	179/140	57/43	122/97	0.903
Primary disease, *n* (%)				0.275
Malignant disease	306 (95.9)	95 (95.0)	211 (96.3)	—
Pancreatic cancer	138 (43.2)	36 (36.0)	102 (46.6)	—
Biliary tract cancer	68 (21.3)	20 (20.0)	48 (21.9)	—
Gastrointestinal cancer	64 (20.1)	25 (25.0)	39 (17.8)	—
Others	36 (11.3)	14 (14.0)	22 (10.0)	—
Benign disease	13 (4.1)	5 (5.0)	8 (3.7)	—
Indications for EUS‐HGS, *n* (%)				0.215
Primary drainage	196 (61.4)	56 (56.0)	140 (63.9)	—
Salvage drainage	123 (38.6)	44 (44.0)	79 (36.1)	—
Ascites, *n* (%)				0.017
<Mild	39 (12.2)	17 (17.0)	22 (10.0)	
≥Moderate	40 (12.5)	18 (18.0)	22 (10.0)	
Ascites drainage before EUS‐HGS, *n* (%)	8 (2.5)	3 (3.0)	5 (2.3)	0.709

Values are presented as median (range) or number (%).

EUS‐HGS, endoscopic ultrasound‐guided hepaticogastrostomy.

Table 2 presents a detailed overview of the EUS‐HGS procedural characteristics. Significant differences were observed between the conventional and enhanced scope groups in the proportion of B2‐only punctures, the reasons for difficulty in B2 puncture, the choice of FNA needle, dilators, and the type of stent deployed (*p* < 0.001). In addition, the presence or absence of an antegrade stent also differed significantly between the two groups (*p* = 0.006). No significant differences were found in B2 bile duct diameter or the endoscopist performing the procedure.

**TABLE 2:** Details of the endoscopic ultrasound‐guided hepaticogastrostomy (EUS‐HGS) procedure.

Characteristics	All (*n* = 319)	Conventional endoscope (*n* = 100)	Enhanced endoscope (*n* = 219)	*p*‐Value
Puncture site, *n* (%)				
B2 alone	247 (77.4)	38 (38.0)	209 (95.4)	< 0.001
The reason for the difficulty of B2 puncture, *n* (%)				< 0.001
Risk of esophageal puncture	30 (9.4)	30 (48.4)	0 (0)	—
Bile duct diameter of B2 was small or no dilation	24 (7.5)	18 (29.0)	6 (60.0)	—
Difficulty inserting the guidewire	14 (4.4)	12 (19.4)	2 (20.0)	—
Vascular puncture, including the portal vein	4 (1.3)	2 (3.2)	2 (20.0)	—
Mean bile duct (B2) diameter (mm), (range)	3.3 (0.9–10.6)	3.8 (1.9–8.4)	3.2 (0.9–10.6)	0.375
Needle, *n* (%)				< 0.001
22‐G	183 (57.4)	23 (23.0)	160 (73.0)	—
19‐G	136 (42.6)	77 (77.0)	59 (27.0)	—
Dilator, *n* (%)				< 0.001
Drill	97 (30.4)	17 (17.0)	80 (36.5)	—
Bougie	107 (33.5)	49 (49.0)	58 (26.5)	—
Balloon	56 (17.6)	23 (23.0)	33 (15.0)	—
Electrocautery	1 (0.3)	1 (1.0)	0 (0)	—
Without	58 (18.2)	10 (10.0)	48 (22.0)	—
Stent, *n* (%)				0.006
6‐mm FCSEMS	240 (75.2)	82 (82.0)	158 (72.1)	—
8‐mm FCSEMS	33 (10.3)	3 (3.0)	30 (13.7)	—
Plastic stent	29 (9.1)	7 (7.0)	22 (10.0)	—
Antegrade stent	40 (12.5)	28 (28.0)	12 (5.5)	< 0.001
Endoscopist, *n* (%)				0.315
Expert	207 (64.9)	69 (69.0)	138 (63.0)	—
Non‐expert	112 (35.1)	31 (31.0)	81 (27.0)	—

Values are presented as median (range) or number (%).

EUS‐HGS, endoscopic ultrasound‐guided hepaticogastrostomy.

FCSEMS, fully coated self‐expandable metal stent.

Table 3A outlines the clinical outcomes associated with the B2‐EUS‐HGS procedures. As shown in Tables 2 and 3A, B2 puncture was achieved in 247 cases, of which 240 underwent successful B2‐EUS‐HGS. The technical/overall clinical success rates by scope type were 35%/91.4% with the GF‐UCT260, 94.3%/96% with the TGF‐UC260J, and 92.9%/98% with the EG‐740UT.

**TABLE 3A:** Clinical outcomes of B2‐EUS‐HGS.

		Conventional endoscope (*n* = 100)	Enhanced endoscope (*n* = 219)	
Characteristics	All (*n* = 319)	GF‐UCT260 (*n* = 100)	TGF‐UC260J (*n* = 106)	EG‐740UT (*n* = 113)
Technical success rate, *n* (%)	240 (75.2)	35 (35.0)	100 (94.3)	105 (92.9)
Overall clinical success rate, *n* (%)	231 (96.3)	32 (91.4)	96 (96.0)	103 (98.0)
Early adverse event rate (<14 days), *n* (%)	15 (4.7)	5 (5.0)	6 (5.7)	4 (3.5)
Early adverse events, *n*, grade				
Abdominal pain	4, mild	2, mild	1, mild	1, mild
Acute cholecystitis	2, moderate	2, moderate	0	0
Bile peritonitis	1, moderate	0	1, moderate	0
Biloma	2, moderate	0	2, moderate	0
Focal cholangitis	4, mild/moderate	1, mild	1, moderate	2, moderate
Internal stent migration	1, moderate	0	0	1, moderate
Bleeding	1, mild	0	1, mild	0
Transesophageal puncture	0	0	0	0

EUS‐HGS: endoscopic ultrasound‐guided hepaticogastrostomy.

All AEs were mild or moderate. This included four cases of mild abdominal pain, two cases of moderate acute cholecystitis, one case of moderate bile peritonitis, two cases of moderate biloma, four cases of focal cholangitis (mild in one case, moderate in three cases), one case of internal stent migration, and one case of mild bleeding.

Four cases of abdominal pain were relieved conservatively. Both cases of moderate acute cholecystitis were later treated with percutaneous transhepatic gallbladder drainage, but hospitalization was prolonged to 10 days. The case of moderate bile peritonitis showed ascites the next day and required drainage, with an FCSEMS (6 mm × 12 cm HANARO Benefit; M.I. Tech., Gyeonggi‐do, Korea) added in the HGS stent. For the two cases of biloma, one additional EUS‐guided abscess drainage was performed, and an FCSEMS was implanted, and the other case underwent EUS‐HGS stent exchange. Two of the patients with moderate focal cholangitis (one with the TGF‐UC260J and one with the EG‐740UT) required HGS stent exchange. In the remaining two cases, the inflammatory response improved with antimicrobial therapy. In one case, scope interference occurred immediately following the deployment of the metal stent, resulting in stent dislocation into the stomach. To manage this complication, the position of the metal stent was adjusted using a duodenoscope. The hemorrhage case was clinically unremarkable, although contrast‐enhanced CT was performed because of a decrease in serum hemoglobin level the day after the procedure. The cause was thought to be accidental portal vein puncture during bile duct puncture. That patient was followed up conservatively and discharged. No cases showed serious AEs, transesophageal puncture, mediastinitis, aspiration pneumonia, or reflux esophagitis, and no deaths occurred.

Table 3B shows the clinical outcomes of B2‐EUS‐HGS comparing the conventional and enhanced endoscope groups. A significant difference was observed between the two groups in terms of technical success rate (*p* < 0.001). There were no statistically significant differences between the two groups in overall clinical success rate (*p* = 0.128) and early adverse event rate (*p* = 0.461). A comparison based on the types of adverse events also showed no significant differences between the two groups.

**TABLE 3B:** Clinical outcomes of B2‐EUS‐HGS (comparison of conventional and enhanced endoscopes).

Characteristics	All (*n* = 319)	Conventional endoscope (*n* = 100)	Enhanced endoscope (*n* = 219)	*p*‐Value
Technical success rate, *n* (%)	240 (75.2)	35 (35.0)	205 (93.6)	<0.001
Overall clinical success rate, *n* (%)	231 (96.3)	32 (91.4)	199 (97.1)	0.128
Early adverse event rate (<14 days), *n* (%)	15 (4.7)	5 (5.0)	10 (4.6)	0.461
Early adverse events, *n*, grade				
Abdominal pain	4, mild	2, mild	2, mild	—
Acute cholecystitis	2, moderate	2, moderate	0	—
Bile peritonitis	1, moderate	0	1, moderate	—
Biloma	2, moderate	0	2, moderate	—
Focal cholangitis	4, mild/moderate	1, mild	3, moderate	—
Internal stent migration	1, moderate	0	1, moderate	—
Bleeding	1, mild	0	1, mild	—
Transesophageal puncture	0	0	0	—

EUS‐HGS: endoscopic ultrasound‐guided hepaticogastrostomy.

As shown in Tables 2 and 3B, B2 puncture using a conventional endoscope was attempted in 38 cases, of which B2‐EUS‐HGS was successful in 35 cases and unsuccessful in three cases. The reasons for failure and subsequent clinical courses of the three unsuccessful cases were as follows:

1. Esophageal puncture occurred, and therefore, HGS stent placement was not performed; hemostatic clipping of the esophagus was carried out, and PTBD was performed later.
2. Although B2 was punctured, guidewire insertion was difficult, and PTBD was subsequently performed.
3. B2 puncture was attempted but was technically difficult; the procedure was converted to ERCP on a later day, and biliary stent placement was performed.

## 4 Discussion

ERCP is sometimes challenging in patients with duodenal stenosis or surgical anatomy. One alternative is EUS‐BD, among which EUS‐HGS has been gaining widespread use since the first report by Burmester et al. [21]. In EUS‐HGS, bile duct‐side stenting site options are B2 and B3. B3 puncture is the most common choice in terms of AEs [11], but B2 puncture may be safer and easier depending on the choice of scope [14, 15, 16]. This study was conducted to assess the technical success and clinical outcomes of B2‐EUS‐HGS performed using three types of EUS scopes: GF‐UCT260, TGF‐UC260J, and EG‐740UT. The three EUS scopes were categorized into two groups: the conventional endoscope group (GF‐UCT260) and the enhanced endoscope group (TGF‐UC260J and EG‐740UT). Regarding the choice of scope, the only reason was the introduction period; there were no factors related to the patients or the procedures. As shown in Table 3B, the technical success and clinical success rates in the enhanced endoscope group were 93.6% and 97.1%, respectively. Previous efficacy evaluations of EUS‐HGS [22, 23, 24, 25] have shown technical success rates of 94%–100% and clinical success rates of 72%–95%, with no difference from the results of the present study. The present results suggest that the enhanced endoscope group may be more suitable for B2 puncture than the conventional endoscope group. To avoid transesophageal puncture and perform puncture of segment B2 from within the stomach, we have also devised a marking clip on the esophagogastric junction [11], and strong up‐angulation is required. In some cases, a nearly vertical puncture angle is needed on the EUS image. Therefore, the degree of up‐angulation is an important performance factor of the scope. However, the GF‐UCT260 has weaker up‐angulation compared with other scopes, making it difficult to achieve a near‐vertical puncture angle and sometimes making puncture of B2 from the stomach challenging. Therefore, the conventional endoscope group may have a lower success rate for B2‐EUS‐HGS, and the enhanced endoscope group may be more suitable for performing B2‐EUS‐HGS.

Based on Tables 3A and 3B, the use of the enhanced endoscope group for B2‐EUS‐HGS was associated with a significantly higher technical and overall clinical success rate compared to the conventional endoscope group. These findings suggest that the enhanced endoscope group may offer potential advantages over the conventional endoscope group in performing B2‐EUS‐HGS.

To avoid transesophageal puncture and perform puncture of segment B2 from within the stomach, strong up‐angulation is required. In some cases, a nearly vertical puncture angle is needed on the EUS image. Therefore, the degree of up‐angulation is an important performance factor of the scope. However, the GF‐UCT260 has weaker up‐angulation compared with other scopes, making it difficult to achieve a near‐vertical puncture angle and sometimes making the puncture of B2 from the stomach challenging.

In the enhanced endoscope group, the TGF‐UC260J has a limited EUS observation range of 90° compared with the EG‐740UT and lacks both an elevator and a locking function. Therefore, it may be difficult to visualize the target bile duct or the vessels to be avoided, and guidewire deviation during exchange may occur. These factors suggest that operators may require some time to become familiar with its use.

Regarding early adverse events, the incidence ranged from 3.5% to 5.7% depending on the type of scope used, with no statistically significant differences observed between the groups. Moreover, no severe adverse events were reported, including esophageal puncture, mediastinitis, aspiration pneumonia, or reflux esophagitis. The overall incidence of adverse events observed in this study was lower than that reported in recent meta‐analyses (the overall incidence of AEs was 15.5%, the incidence of serious AEs was 0.6%, and the procedural mortality rate was 0.2%) [26], indicating that B2‐EUS‐HGS using any of the three scopes may be a safe procedure. Along with our previous reports [15, 16], this study compared three EUS scopes with a conventional OV scope, but because our study was retrospective in design, these facts need to be confirmed in further studies.

This study has several limitations, including its retrospective, non‐randomized, single‐center design, potential selection bias in the choice of FNA needles, and the possible contribution of a novel device (drill dilator) to the procedural success rate; therefore, the results should be evaluated by a prospective, randomized trial.

In conclusion, enhanced endoscopes may be useful when attempting B2‐EUS‐HGS.

## Author Contributions

**Yoshitaro Yamamoto**: conceptualization; data curation; formal analysis; methodology; writing – original draft; writing – review and editing. **Kazuo Hara**: conceptualization; formal analysis; methodology; writing – original draft; writing – review and editing. **Nozomi Okuno**: data curation; formal analysis; writing – original draft; writing – review and editing. **Shin Haba**: data curation; writing – review and editing. **Takamichi Kuwahara**: Data curation; writing – review and editing. **Hiroki Koda**: data curation; writing – review and editing. **Minako Urata**: Data curation; writing – review and editing. **Takashi Kondo**: data curation; writing – review and editing. **Keigo Oshiro**: data curation; writing – review and editing. **Tomoki Ogata**: data curation; writing – review and editing. **Ren Kuwabara**: data curation; writing – review and editing.

## Conflicts of Interest

Kazuo Hara has received lecture fees from FUJI FILM, OLYMPUS, Boston Scientific, and Asahi Intecc. The other authors declare no conflicts of interest.

## Funding

The authors received no specific funding for this work.

## Ethics Statement

All patients provided informed consent for the procedures, and the study protocol was approved by the institutional review board of Aichi Cancer Center Hospital (approval no. 2024‐0‐366).

## References

[1] B. R. Boulay and S. K. Lo ., “Endoscopic Ultrasound‐guided Biliary Drainage,” Gastrointestinal Endoscopy Clinics of North America 28 (2018): 171–185. 29519330 10.1016/j.giec.2017.11.005

[2] A. Y. Teoh , V. Dhir , M. Kida , et al., “Consensus Guidelines on the Optimal Management in Interventional EUS Procedures: Results From the Asian EUS Group RAND/UCLA Expert Panel,” Gut 67 (2018): 1209–1228. 29463614 10.1136/gutjnl-2017-314341

[3] V. Dhir , H. Isayama , T. Itoi , et al., “Endoscopic Ultrasonography‐guided Biliary and Pancreatic Duct Interventions,” Digestive Endoscopy 29 (2017): 472–485. 28118509 10.1111/den.12818

[4] N. Okuno , K. Hara , N. Mizuno , et al., “Efficacy of the 6Mm Fully Covered Self‐expandable Metal Stent During Endoscopic Ultrasound‐guided Hepaticogastrostomy as a Primary Biliary Drainage for the Cases Estimated Difficult Endoscopic Retrograde Cholangiopancreatography: A Prospective Clinical Study,” Journal of Gastroenterology and Hepatology 33 (2018): 1413–1421. 29424011 10.1111/jgh.14112

[5] D. Oh , D. H. Park , T. J. Song , et al., “Optimal Biliary Access Point and Learning Curve for Endoscopic Ultrasound‐guided Hepaticogastrostomy With Transmural Stenting,” Therapeutic Advances in Gastroenterology 10 (2017): 42–53. 28286558 10.1177/1756283X16671671 PMC5330611

[6] J. J. Vila , M. PérezMiranda , E. VazquezSequeiros , et al., “Initial Experience With EUS‐guided Cholangiopancreatography for Biliary and Pancreatic Duct Drainage: A Spanish National Survey,” Gastrointestinal Endoscopy 76 (2012): 1133–1141. 23021167 10.1016/j.gie.2012.08.001

[7] K. Hara , K. Yamao , N. Mizuno , et al., “Endoscopic Ultrasonography‐guided Biliary Drainage: Who, When, Which, and How?” World Journal of Gastroenterology 22 (2016): 1297–1303. 26811666 10.3748/wjg.v22.i3.1297 PMC4716039

[8] W. H. Paik and D. H. Park ., “Outcomes and Limitations: EUS‐guided Hepaticogastrostomy,” Endoscopic Ultrasound 8 (2019): S44–S49. 31897379 10.4103/eus.eus_51_19 PMC6896431

[9] S. Matsumoto , K. Hara , N. Mizuno , et al., “Risk Factor Analysis for Adverse Events and Stent Dysfunction of Endoscopic Ultrasound‐guided Choledochoduodenostomy,” Digestive Endoscopy 32 (2020): 957–966. 31883405 10.1111/den.13620

[10] H. Isayama , Y. Nakai , T. Itoi , et al., “Clinical Practice Guidelines for Safe Performance of Endoscopic Ultrasound/Ultrasonography‐guided Biliary Drainage: 2018,” Journal of Hepato‐Biliary‐Pancreatic Sciences 26 (2019): 249–269. 31025816 10.1002/jhbp.631 PMC7064894

[11] N. Okuno , K. Hara , N. Mizuno , et al., “Risks of Transesophageal Endoscopic Ultrasonography‐guided Biliary Drainage,” Gastrointestinal Intervention 6 (2017): 82–84.

[12] T. Ogura and K. Higuchi ., “Technical Tips for Endoscopic Ultrasound‐guided Hepaticogastrostomy,” World Journal of Gastroenterology 22 (2016): 3945–3951. 27099437 10.3748/wjg.v22.i15.3945 PMC4823244

[13] T. Ogura and K. Higuchi ., “Endoscopic Ultrasound‐guided Hepaticogastrostomy: Technical Review and Tips to Prevent Adverse Events,” Gut Liver 15 (2021): 196–205. 32694240 10.5009/gnl20096 PMC7960972

[14] K. Hara , N. Okuno , S. Haba , et al., “How to Perform EUS‐guided Hepaticogastrostomy Easier and Safer,” Journal of Hepato‐Biliary‐Pancreatic Sciences 27 (2020): 563–564. 32511837 10.1002/jhbp.774

[15] N. Okuno , K. Hara , N. Mizuno , et al., “B2 puncture With Forward‐viewing EUS Simplifies EUS‐guided Hepaticogastrostomy (With Video),” Endoscopic Ultrasound 11 (2022): 319–324. 35848655 10.4103/EUS-D-21-00154 PMC9526104

[16] S. Ishikawa , N. Okuno , K. Hara , et al., “Safety and Efficacy of Novel Oblique‐viewing Scope for B2‐Endoscopic Ultrasound‐guided Hepaticogastrostomy,” Clinical Endoscopy 57, no. 4 (2024): 527–533, 10.5946/ce.2023.129. 38549245 PMC11294849

[17] N. Okuno , K. Hara , N. Mizuno , et al., “Infectious Peritonitis After Endoscopic Ultrasound‐guided Biliary Drainage in a Patient With Ascites,” Gastrointestinal Intervention 7 (2018): 40–43.

[18] K. Hara , K. Yamao , N. Mizuno , et al., “Endoscopic Ultrasonography‐guided Biliary Drainage: Who, When, Which, and How?” World Journal of Gastroenterology 22 (2016): 1297–1303. 26811666 10.3748/wjg.v22.i3.1297 PMC4716039

[19] H. Isayama , T. Hamada , T. Fujisawa , et al., “TOKYO Criteria 2024 for the Assessment of Clinical Outcomes of Endoscopic Biliary Drainage,” Digestive Endoscopy 36 (2024): 1195–1210. 38845085 10.1111/den.14825

[20] P. B. Cotton , G. M. Eisen , L. Aabakken , et al., “A Lexicon for Endoscopic Adverse Events: Report of an ASGE Workshop,” Gastrointestinal Endoscopy 71 (2010): 446–454. 20189503 10.1016/j.gie.2009.10.027

[21] E. Burmester , J. Niehaus , T. Leineweber , et al., “EUS Cholangiodrainage of the Bile Duct: Report of 4 Cases,” Gastrointestinal Endoscopy 57 (2003): 246–251. 12556796 10.1067/mge.2003.85

[22] A. Anderloni , A. Fugazza , M. Spadaccini , et al., “Feasibility and Safety of a New Dedicated Biliary Stent for EUS‐guided Hepaticogastrostomy: The FIT Study (With Video),” Endoscopic Ultrasound 12 (2023): 59–63. 36510880 10.4103/EUS-D-22-00023 PMC10134938

[23] M. Jagielski , M. Zielinski , J. Piatkowski , et al., “Outcomes and Limitations of Endoscopic Ultrasound‐guided Hepaticogastrostomy in Malignant Biliary Obstruction,” BMC Gastroenterology [Electronic Resource] 21 (2021): 202. 33952187 10.1186/s12876-021-01798-2 PMC8097803

[24] F. Moryoussef , A. Sportes , S. Leblanc , et al., “Is EUS‐guided Drainage a Suitable Alternative Technique in Case of Proximal Biliary Obstruction?” Therapeutic Advances in Gastroenterology 10 (2017): 537–544. 28804514 10.1177/1756283X17702614 PMC5484435

[25] N. Okuno , K. Hara , N. Mizuno , et al., “Efficacy of the 6‐mm Fully Covered Self‐Expandable Metal Stent During Endoscopic Ultrasound‐guided Hepaticogastrostomy as a Primary Biliary Drainage for the Cases Estimated Difficult Endoscopic Retrograde Cholangiopancreatography: A Prospective Clinical Study,” Journal of Gastroenterology and Hepatology 33 (2018): 1413–1421. 29424011 10.1111/jgh.14112

[26] S. Giri , B. P. Mohan , V. Jearth , et al., “Adverse Events With EUS‐guided Biliary Drainage: A Systematic Review and Meta‐analysis,” Gastrointestinal Endoscopy 98 (2023): 515–523. e8. 37392952 10.1016/j.gie.2023.06.055

